# Effective Endovascular Stenting of Malignant Portal Vein Obstruction in Pancreatic Cancer

**DOI:** 10.1155/2009/426436

**Published:** 2009-10-11

**Authors:** Christian M. Ellis, Sadashiv Shenoy, Alan Litwin, Stephanie Soehnlein, John F. Gibbs

**Affiliations:** Roswell Park Cancer Institute, State University of New York at Buffalo, Buffalo, NY 14263, USA

## Abstract

We report herein the case of a patient successfully treated by transhepatic portal venous stent placement for malignant portal vein obstruction with associated gastric and small bowel varices and repeated gastrointestinal bleeding. CT angiography and portography showed severe portal vein obstruction from recurrent pancreatic cancer 15 months following pancreaticoduodenectomy with tumor encasement and dilated collateral veins throughout the gastric and proximal small bowel wall as the suspected cause of the GI bleeding. Successful transhepatic endovascular stent placement of the splenic vein at the portal vein confluence followed by balloon dilation was performed with immediate decompression of the gastric and small bowel varices and relief of GI hemorrhage in this patient until his death four months later. The treatment for patients with this dilemma can prove to be difficult, but as we have shown endovascular stenting of the portal system is an effective treatment option.

## 1. Introduction

Pancreatic cancer remains a major oncologic challenge making up 2% of all new cancer cases and 6% of cancer deaths in the United States [1]. Although some cancer centers report up to 20% 5-year survival rate after pancreaticoduodenectomy, the outlook, morbidity, and mortality for this disease remain bleak [2]. Therefore, in patients who develop recurrent metastatic disease, new methods of treatment and palliation are necessary to improve their quality of life. In cases of malignant portal venous obstruction from tumor, such as in hepatocellular carcinoma, cholangiocarcinoma, and pancreatic cancer endovascular stents have demonstrated the ability to relieve symptoms associated with this type of presentation [3]. We report the case of a patient after pancreaticoduodenectomy who developed near complete splenic vein obstruction with symptomatic gastric and small bowel mesenteric varices that was revascularized by transhepatic placement of an endovascular stent into the splenic vein (SV) at the portal vein (PV) confluence.

## 2. Case Report

A 58-year-old man was referred to our institution for a suspicious mass within the neck of the pancreas with a dilated pancreatic duct and evidence of chronic pancreatitis identified by abdominal computed tomography (CT). An endoscopic ultrasound (EUS) and endoscopic retrograde cholangiopancreatography (ERCP) identified adult diagnosed pancreatic divisum with a 2.5 cm mass within the neck of the pancreas and pancreatic duct stricture. A fine needle aspiration (FNA) of this area showed only chronic inflammation and a CA 19-9 level returned within normal limits. Review at our institutional multidisciplinary conference led to recommendations for surgical resection, and ten months after his initial presenting symptoms, the patient underwent a pylorus-preserving pancreaticoduodenectomy. The final pathology revealed a specimen with chronic pancreatitis and one small focus of invasive carcinoma with perineural invasion. No tumor was identified in twenty-one lymph nodes. All surgical margins were negative for tumor. The patient made an uneventful recovery and afterward elected to forego any further adjuvant treatments. 

The patient did well while undergoing routine surveillance until approximately 15 months later when he began to experience mild to moderate abdominal discomfort and nausea. A repeat CT scan of the abdomen revealed a mass at the site of the previous surgical resection with narrowing of the PV confluence and extensive lymphadenopathy at the root of the mesentery. An ultrasound-guided biopsy of this area confirmed recurrent well-differentiated adenocarcinoma, and the patient began concurrent chemoradiotherapy with continuous infusion 5-fluorouracil and external beam radiation therapy (EBRT) for a total dose of 54 Gy. 

 On follow-up the patient developed upper gastrointestinal (GI) bleeding, requiring multiple blood transfusions, and on upper endoscopy was found to be due to varices of the proximal gastric wall, which appeared to extended through the anastamosis into the efferent jejunal limb. At the time of endoscopy there was no evidence of active bleeding, and so we elected to proceed with medical management for the patient to include *β*-blockers and intravenous octreotide. 

**Figure 1:**
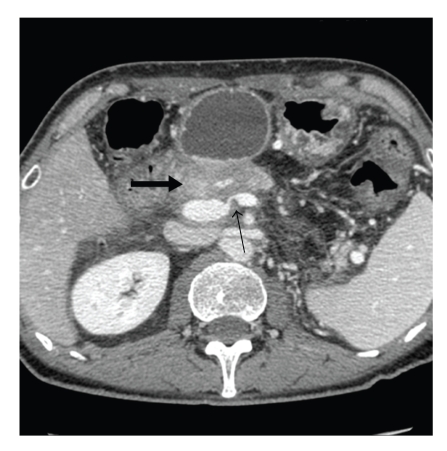
CT evidence of the recurrent pancreatic mass in the previous surgical bed (large arrow) and demonstration of obstruction of the splenic vein at the portal vein confluence (small arrow).

**Figure 2:**
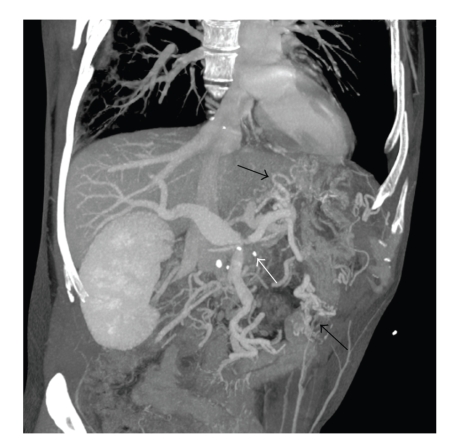
CT angiography coronal plane reconstruction showing the portal vein system with obstruction from the recurrent pancreatic tumor at the portal vein confluence (white arrow) and resulting gastric and small bowel varices (black arrows).

**Figure 3:**
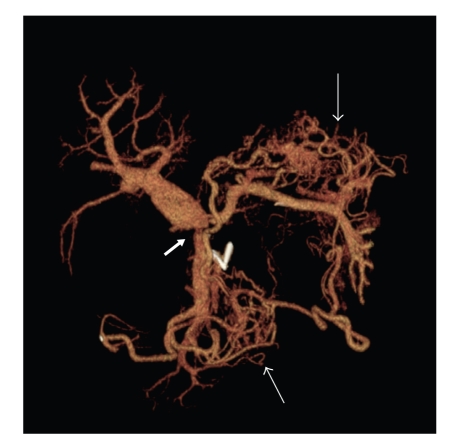
Isolated portal system reconstruction defining the obstruction of the splenic vein at the portal vein confluence (block arrow) and clear confirmation of the subsequent gastric and small bowel varices (normal arrows).

**Figure 4:**
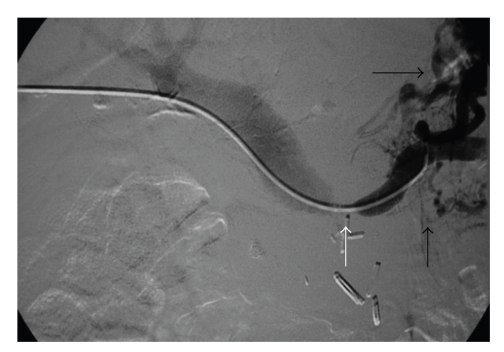
Transhepatic portography characterizing the splenic vein obstruction from the recurrent pancreatic tumor (white arrow) with dilated varices proximally (black arrows).

**Figure 5:**
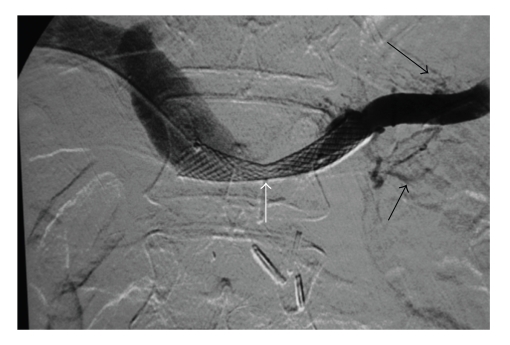
Transhepatic portography after endovascular stent placement and balloon dilation of the splenic vein obstruction (white arrow) with dramatic decompression of the proximal varices (black arrows).

During the same hospitalization the patient again developed variceal bleeding. We found that the recurrent pancreatic tumor had caused a high-grade neoplastic obstruction of the SV at the PV confluence and as a consequence resulted in mesenteric hypertension and gastric and small bowel varices that were refractory to our medical management (Figures 1, 2, and 3). Because of this treatment dilemma we sought the expertise of our interventional radiology department who confirmed the SV obstruction and dilated varices by transhepatic portography (Figure 4). They were then able to successfully treat the patient by transhepatic endovascular stenting of the SV obstruction with a 10 mm × 42 mm wall stent followed by an 8 mm balloon dilation. Repeat splenic venography demonstrated the PV confluence to be patent with good flow across the stent along with dramatic decompression of the collateral gastric and small bowel varices (Figure 5).

Immediately after the procedure the patient had no additional episodes of melena or hematemesis, and no further blood transfusions were required. Seven days following the procedure the patient was discharged home from the hospital tolerating an advancing diet. He died 4 months later from natural progression of the malignancy. During this time, he did not experience any additional episodes of GI bleeding and remained relatively comfortable.

## 3. Discussion

Pancreatic cancer remains an oncologic challenge where early metastatic relapse after complete resection is frequently encountered. Sperti et al. reported local and hepatic recurrence rates of 72% and 62%, respectively, in patients undergoing curative resection [4]. In the current study, our patient presented with a 2.5 cm mass arising in the background of pancreatic divisum and chronic pancreatitis. On final pathology, he was found to have Stage I T1N0 disease. The only poor prognostic factor identified was perineural involvement. In a recent review, perineural invasion was noted in 70% of patients and was associated with a significantly poorer median disease free survival of 16.2 months [5]. We believe that the recurrence noted in our patient involved the extrapancreatic nerve plexus [6]. Alternatively, the site of recurrence may be argued secondary to occult multifocal pancreatic cancer. The lack of pancreatic intraepithelial neoplasia within the resected specimen and numerous follow-up imaging is against the later argument.

Extrahepatic portal venous obstruction accounts for 5%–10% of all cases of portal hypertension, with neoplasms such as hepatocellular carcinoma and pancreatic and biliary cancer accounting for 15%–24% of those cases [3]. Cancer can lead to thrombosis of the portal venous system through a combination of factors including cancer-related prothrombogenic changes, tumor invasion, periportal fibrosis following surgery or radiotherapy, or more commonly from extrinsic compression or constriction from tumor mass [7, 8]. Other causes of portal hypertension from extrahepatic occlusion or thrombosis include infection complicated by peritonitis, liver abscess, biliary tract surgery, and congenital abnormalities [9]. Coagulopathies that lead to thrombosis and thrombophlebitis migrans can also be a cause [9].

Prehepatic portal hypertension from portal vein stenosis or occlusion secondary to malignant invasion is a difficult entity to diagnose and treat. This difficulty is exemplified by the fact that occlusion of the portal vein frequently does not produce an acute manifestation. The reasons are twofold why the blocking of portal blood flow, which accounts for two thirds of the total hepatic supply, results in few clinical manifestations. The first is because of the compensatory mechanism of vasodilation of the hepatic arterial system occurs in response to a decrease in portal vein flow [10]. The second is a process termed *cavernous transformation*, for which is the rapid development of tortuous collateral veins bypassing the thombosed or occluded portion of the portal vein that will usually become apparent within a matter of days [10]. Herein lies the manifestation most devastating to patients with portal venous obstruction.

When symptoms do occur, they can present in a variety of ways [8, 10–12]. The most frequent manifestation resulting in patient's seeking medical attention is hematemesis or melena from the development of varices in the esophagus, stomach, small intestine, and colon, which can result in severe gastrointestinal bleeding and ultimately death [8, 11]. Janssen et al. retrospectively evaluated 172 patients (27% of patients had hepatobiliary or gastrointestinal malignancies) with extrahepatic portal vein thrombosis and found that an episode of bleeding from ruptured esophagogastric varices was the initial manifestation in 52 (30%) patients [12]. Furthermore, of the 130 patients tested, 104 patients demonstrated esophageal varices and 4 percent of those patients died from their variceal hemorrhage [12].

As exemplified by our patient, these cases can be a diagnostic and treatment dilemma, with treatment options being few due to rapid tumor growth and related diseases resulting in a very poor prognosis. Historically, treatments for such conditions have been radiation therapy or chemotherapy; however, resolution of the signs and symptoms may have a delayed period of effect up to 3 weeks [13]. These patients are typically not good surgical candidates due to their poor clinical status and therefore are in need of less invasive methods for palliating their symptoms. Endoscopic variceal sclerotherapy has been effective in treating varices of the esophagus and stomach but are ineffective for bleeding further down the gastrointestinal tract. By applying the same physiology of esophagogastric varices to the small bowel, some of these patients can be treated medically with octreotide, propanolol, and other forms of therapy such as chemoradiotherapy [14]. Even with these conservative therapies patients still require multiple hospitalizations from associated symptoms and suffer from prolonged episodes of bleeding, with always the risk of sudden death. As physicians treating cancer we should always seek ways in which to palliate our patients with as little disability as possible, and as shown with our patient percutaneous placed endovascular stents has permitted our ability to effectively treat these patients with this type of condition.

We report here the successful SV at the PV confluence stenting of a patient with active variceal bleeding due to recurrent pancreatic cancer. A MEDLINE review of literature yields a total of 64 reported cases of portal vein stent placement, the first of which was by Harville et al. in 1991 [15]. They were able to establish portal vein patency and relief of massive lower GI hemorrhage from colonic varices in a patient with portal hypertension caused by extrahepatic obstruction from chronic pancreatitis. Mathias et al. demonstrated successful use of this technique in a patient with portal venous stenosis due to encasement by pancreatic cancer resulting in portal venous hypertension and variceal bleeding [16]. Portal hypertension was relieved with no recurrent variceal bleeding during the 5 months before the patient died [16]. Watanabe et al. have shown in two patients with malignant portal stenosis that metallic stent implantation can reduce portal pressures dramatically resulting in normalization of liver function tests and reduction of ascites [13].

The largest series reported in literature is by Yamakodo et al. where they were able to place stents into the portal venous system via a percutaneous transhepatic route across 28 stenotic and 12 obstructive lesions [3]. They reported a mean followup period of 11.9 months (range, 2–61 months), during which 60% percent of the stents remained patent [3]. Stent occlusion was found in 40% of the patients, with a mean period until stent occlusion of 3.7 months (range, 0.2–16 months) clearly showing an effective method of treatment in patients with short life expectancies [3].

Extrahepatic portal venous obstruction can be a major cause of portal hypertension and morbidity to these patients. It seems that in a selected patient population portal venous stents can be used affectively to palliate the symptoms caused by portal hypertension due to these types of conditions. Future studies, like the one by Yamakodo et al. showing that portal venous stent patency is prolonged when there is not substantial splanchnic vein involvement, are needed to further clarify and classify which group of patients with malignant portal venous involvement would benefit from such therapy [11]. At this time, it seems appropriate to consider stent placement when surgery is contraindicated, life expectancy is short, or other conservative methods of treatment have been exhausted. Portal venous stent placement provides rapid decompression of varices and palliation of symptoms with little morbidity to the patient helping to improve their quality of life and prognosis. We feel that it is important for physicians to be aware of the tools available for palliation and add them to their armitarium for the care of patients with such complex problems and devastating diseases.

The presence of recurrence occurring at the superior mesenteric/portal venous confluence raises the question of initial portal vein resection. Although the indication and contraindication for portal vein resection has not universally defined, there are many reports addressing its benefit [6, 17–19]. The MD Anderson groups, who are known advocates of portal vein resection, have recently reported that this patient population have a higher likelihood of an R1 resection [20].

## 4. Conclusion

The treatment for patients with malignant superior mesenteric/portal vein obstruction and associated bleeding esophageal and gastric varies can prove to be difficult, but as we have shown endovascular stenting of the portal system is an effective treatment option.
